# A Model for Implementing Psychotherapy for Individuals with Cognitive Impairments

**DOI:** 10.1002/alz.091331

**Published:** 2025-01-09

**Authors:** Adrienne L Johnson, Mary F. Wyman, Nathaniel A. Chin

**Affiliations:** ^1^ University of Wisconsin School of Medicine and Public Health, Madison, WI USA; ^2^ University of Wisconsin ‐ Center for Tobacco Research and Intervention, Madison, WI USA; ^3^ Wisconsin Alzheimer’s Disease Research Center, University of Wisconsin, School of Medicine and Public Health, Madison, WI USA; ^4^ Madison VA GRECC, William S. Middleton Memorial Hospital, Madison, WI USA; ^5^ Wisconsin Alzheimer’s Disease Research Center, Madison, WI USA

## Abstract

Individuals with Mild Cognitive Impairment (MCI) and dementia are at greater risk for mental health conditions including depression and anxiety than those without cognitive impairments.^1,2^ Non‐pharmacological interventions are considered first‐line to address these problems, but access to psychotherapy services for these groups remains limited. Adapted evidence‐based therapy approaches, including Cognitive Behavioral Therapy (CBT)^3‐5^ and Acceptance and Commitment Therapy (ACT)^6^ are effective for treating comorbid mental health conditions in populations with cognitive impairments. We report on the implementation of psychotherapy services for patients with MCI and Mild Dementia within the Memory Assessment Clinic of a large academic healthcare system in Midwest U.S. Pre‐implementation process included identifying a clinic champion to support adoption and observing current clinic flow by the practitioner (a licensed Clinical Health Psychologist with expertise treating patients with neurological issues) to determine logistics. Referrals were initially solely provided by the clinic champion, then opened to the entire clinic with pre‐determined eligibility guidelines: recent MCI or Mild Dementia diagnosis, comorbid anxiety or depressive symptoms, and lack of rapid forgetting in during medical examination. Therapy patients were seen on a short‐term basis (6‐8, 45‐minute sessions) to maximize access for as many patients as possible, using CBT or ACT‐based therapy modalities. Therapy content was tailored and person‐centered but included (in chronological order): evaluation of cognitive capacities to leverage during therapy, identification of treatment goals, identification of problematic cognitions/behaviors, introduction of new treatment content/coping skills (one topic/session), assign homework assignments using learned skills, evaluation of homework effectiveness and roadblocks. The initial session was in‐person to facilitate comprehensive evaluation and build rapport, and family caregivers were included as indicated. Follow‐up sessions were provided in‐person or via telehealth. Barriers to implementation included incorrect use of referral process, lack of provider knowledge of new services, and limited provider availability. Facilitators included developing a wait list for referred patients and regular education to providers with written details of the service (e.g., referral process and eligibility; waitlist duration). Similar strategies have been used in other health systems and suggest the feasibility of incorporating psychological treatment for cognitively impaired patients as part of a multi‐component treatment model.

